# Chasing the Zebra: a case of membranous-like Glomerulopathy with SSA/RO52 deposits and no overt connective tissue disease

**DOI:** 10.1186/s41927-023-00330-1

**Published:** 2023-04-05

**Authors:** Savino Sciascia, Paolo Miraglia, Massimo Radin, Manuela Giarin, Nicolas Charbonier, Antonella Barreca, Irene Cecchi, Irene Lanzetta, Roberta Fenoglio, Elisa Menegatti, Dario Roccatello

**Affiliations:** 1 University Center of Excellence on Nephrologic, Rheumatologic and Rare Diseases (ERK-net, ERN-Reconnect and RITA-ERN Member) with Nephrology and Dialysis Unit, Center of Immuno-Rheumatology and Rare Diseases (CMID), Coordinating Center of the Interregional Network for Rare Diseases of Piedmont and Aosta Valley (North-West Italy), San Giovanni Bosco Hub Hospital, Piazza del Donatore di Sangue 3, 10154 Turin, Italy; 2 Department of Clinical and Biological Sciences, University of Turin, 10154 Turin, Italy; 3 School of Specialization of Clinical Pathology, Department of Clinical and Biological Sciences, University of Turin, Turin, Italy; 4 Pathology Unit, ‘’Città della Salute e della Scienza di Torino’’ University Hospital, Turin, Italy

**Keywords:** Membranous Glomerulonephritis and Masked IgG-Kappa deposits, SSA, Autoantibodies, Membranous nephropathy

## Abstract

**Background:**

The nature of the deposits in immune-mediated glomerulonephritis with a membranous pattern and masked IgG-Kappa deposits (MGMID) remains still to be elucidated.

**Case presentation:**

We present a case of 33-year-old woman developing a continuous asymptomatic proteinuria (0.8–1 g/24 h) with no overt connective tissue diseases. She tested positive at high titers for SSA antibodies (Ro52 838 UI/mL, Ro60 2716 UI/mL) and at the kidney biopsy histological findings were compatible with an immune-mediated glomerulonephritis with a membranous pattern and masked IgG-Kappa deposits. Also, we demonstrated a positive immunohistochemistry staining for anti-Ro52-SSA antibodies, with a granular positivity in mesangium and along rare glomerular capillaries. To date, only one case of a patient with overt diagnosis of Sjögren’s syndrome with MGMID has been described but a pathogenic role for SSA and SSB antibodies has never been proven.

**Conclusions:**

In this case, we described for the first time by immunohistochemistry a Ro52+ granular positivity in the mesangium and glomerular capillaries, potentially paving the way for a better understanding of MGMID.

## Background

In 2014 Larsen et al. [1] first described a new histological entity of membranous nephropathy (MN): the membranous-like glomerulopathy with masked IgG kappa deposits pattern (MGMID). The main characteristic of this condition relies on the fact that glomerular immune complex depositions are masked deposits requiring an antigen-retrieval step to be visualized. In fact, when diagnosing this condition one should remember that performing immunofluorescence staining on frozen tissue may lead to a false negative staining of immunoglobulin since only C3 staining may be detected while the immunoglobulin remains “masked”. When the procedure is repeated on paraffin-embedded tissue after protease digestion the subepithelial immunoglobulin IgG kappa deposits are revealed [2].

This deposition of IgG kappa by protease digestion seem to most commonly occur in young females (< 40 years age) that are positive to autoimmune serologic evaluation including the search of antinuclear antibodies (ANA) [3]; however, the majority of patients with MGMID present with no overt underlying connective tissue diseases. While MN is a common diagnosis among patients with adult nephrotic syndrome, understanding the etiology of MGMID is still challenging, also due to the limited understanding we have of this condition. Recently serum amiloid P (SAP) presence in glomeruli biopsy was associated with MGMID [4].

Herewith, we described a case of a young woman with MGMID with immunohistochemistry evidence of SSA granular positivity in mesangium glomerular capillaries for SSA/RO52 consistent with her serological positivity for high titer SSA antibodies. At best of our knowledge, our case represents the first attempt in characterizing the nature of the deposits in MGMID.

## Case presentation

The patient was a nulliparous 33-year-old caucasian woman with a familiarity for rheumatic diseases (mother with rheumatoid arthritis) but no prior overt rheumatic diseases. During a routine laboratory evaluation she was found to have an asymptomatic proteinuria (58 mg/dl) confirmed in consequent investigations over 6 months time with persistently increased proteinuria (0.8–1 g/24 h) with normal renal function.

When she presented at our center, a full set of investigations was performed. Patient’s laboratory investigations are summed up in Table 1. Laboratory, clinical and instrumental findings were unremarkable, except for the known proteinuria in subnephrotic range and ANA positivity with high titers of SSA/60 and SSA/52 (EliA™ Autoimmunity Testing Solutions, Thermo Fisher Scientific, Waltham, Massachusetts, USA). Taking into account the antibody positivity, the young age and the finding of an isolated but persistent urinary abnormality in a woman with pregnancy prospects, a renal biopsy was performed.

**Table 1:** Laboratory investigations performed at the time of the kidney biopsy

Parameter	Value
Blood pressure	110/60 mmHg
Hb	11,9 g/dl
WBC	7100/mm^3^
Lymphocyte	1770/mm^3^
Platelets	244,000/ mm^3^
Total Cholesterol	172 mg/dL
Triglyceride	73 mg/dL
Urea nitrogen	18 mg/dL
Serum creatinine	0,7 mg/dL
Clearance creatinine	107 cc/min
Serum Calcium	2,4 mmol/dL
Serum Sodium	140 mEq/L
Serum Potassium	4 mEq/L
Serum Phosphate	3,5 mg/dL
Serum IgG	1316 mg/dL
Serum IgA	104 mg/dL
Serum IgM	125 mg/dL
C3	101 mg/dL
C4	15 mg/dL
Total serum protein*	7,1 mg/dL
Albumin	62,1%
Alpha 1 globulins	3%
Alpha 2 globulins	10,5%
Beta 1 globulins	4,9%
Beta 2 globulins	4,1%
Gamma Globulins	15,4%
ANA	1:160
ANA Pattern	Speckled
Anti-SSA(Ro52)	838 UI/dL
Anti-SSA(Ro60)	2716 UI/dL
Anti-SSB	0 UI/dL
Anti-SM	0 UI/dL
Serum cryoglobulins	negative
Urine pH	6.0
24 h proteinuria	1.01 g/24 h
Urinary sediment	No microscopic haematuria
Urine protein electrophoresis immunofixation	Negative

^*^ No abnormalities at the visual inspection of serum protein electrophoresis

*ANA* Anti-nuclear antibody, *Anti-SM* Anti-Smith antibody, *Anti-SSA/B* Anti–Sjögren's-syndrome-related antigen A/B, *C3 / C4* Complement component ¾, *WBC* White blood count, *Hb* Hemoglobin, *Ig* Immunoglobulin

## Kidney biopsy

**Fig. 1:**
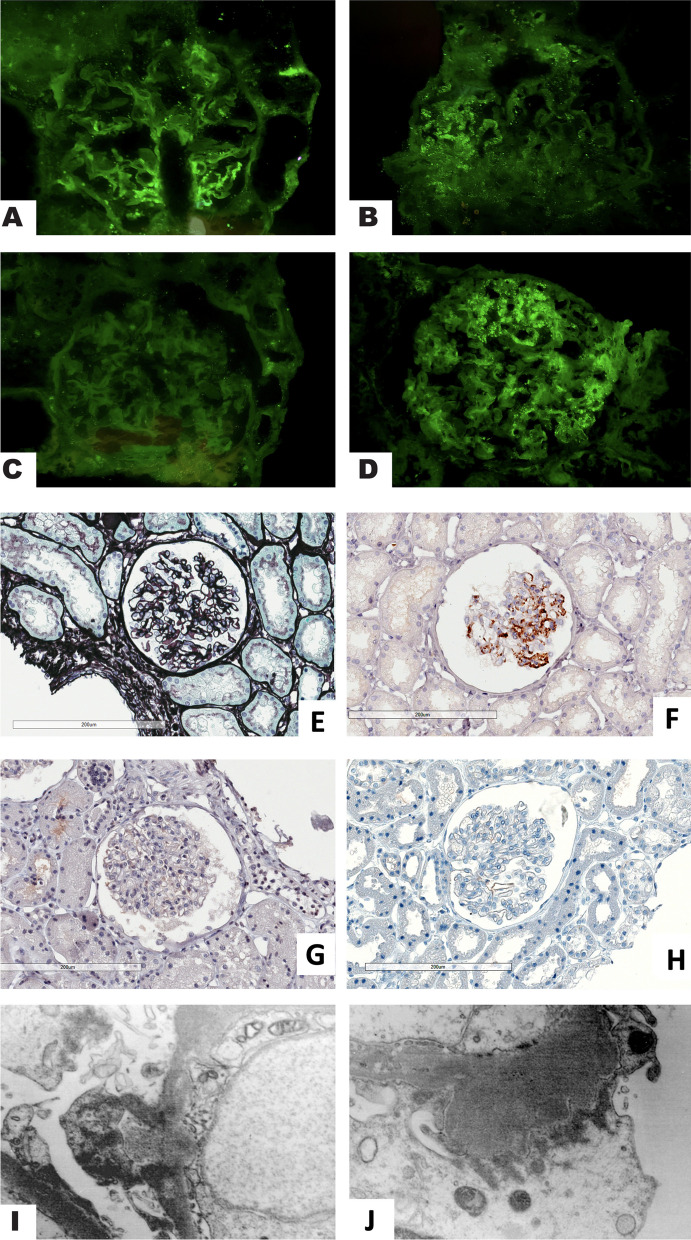
Renal biopsy findings in membranous-like glomerulopathy with masked IgG kappa deposits. Immunofluorescence on fresh tissue showed irregular moderate granular staining for IgG (**A**) along the glomerular capillaries only in limited basement membranes and rare mesangial deposits. Glomeruli revealed positivity for only kappa light chain (**B**) and not for lambda (**C**) (**A**–**C** direct immunofluorescence on fresh material, original magnification × 400). Serum amyloid P (SAP) demonstrated stronger and more diffuse staining in glomeruli with a similar granular pattern (**D**, direct immunofluorescence on fresh tissue, original magnification X 400). **E** Occasional spikes and holes were evident along the glomerular basement membranes by Jones methanamine silver stain (original magnification × 200; scanner Hamamatsu). **F** Immunohistochemistry on fixed material with anti-human C4d antibody demonstrated strong granular positivity along glomerular capillary walls with a diffuse but irregular distribution and occasional mesangial deposits (original magnification × 200; scanner Hamamatsu). Glomeruli were negative both for PLA2R (**G**, original magnification × 200; scanner Hamamatsu) and THSD7A (**H**, original magnification × 200; scanner Hamamatsu). **I**, **J**) Electron microscopy demonstrated sparse amorphous subepithelial deposits along the glomerular basement membrane with occasional spikes and rare mesangial deposits (I,J original magnification × 9800. TEM Philips CM10)

The specimen included 11 corpuscles, one of which with global sclerosis of the floc. The remaining glomeruli have free urinary spaces and diffused slightly thickened basal membranes with a stiff or occasionally irregular appearance. Sections for light microscopy were stained with Phosphotungstic acid haematoxylin (PTAH) and Apigenin (APG), and shows some deposits located in the basal membranes in the sub-epithelial area and, less frequently, the mesangial axes were observable (shown in Fig. 1). The interstitial was made by finely fibrous connective tissue, with only occasional minimal infiltration of by rare small lymphocytes and plasma cells and very rare isolated atrophic tubules. Stain with Congo Red for the amyloid substance was negative. The immunohistochemical investigation carried out on material fixed with anti C4d antibodies was positive (+ + +), with an irregular granular distribution in some sections of the glomerular basal membrane, in the subepithelial area and in some mesangial axes. Immuno-staining was also performed on material fixed with antibodies to phospholipase A2 receptor (PLA2R) with negative result. Immunofluorescence performed on frozen material was positive for IgG (+ +) and mild for C3 ( +) with focal very irregular distribution in limited segments of the basal membrane and in fine granules in the sub-epithelial area. Immunofluorescence was also performed on frozen material with antibodies to a) Kappa and lambda light chains (tested with two different clones) which showed a positive pattern consistent with the IgG for the kappa light chains (+ +), with negative lambda stain; b) P component of the amyloid substance that gave a positive result (+ + +), with a pattern similar to that of IgG but with a more extensive distribution. The histological finding was compatible with an immune-mediated glomerulonephritis with a membranous pattern and masked IgG-Kappa deposits (MGMID).

She was managed with ACE-inhibitors with no further additional therapies, including no immunosuppression. After a follow-up of 24 months, she presented with no further sign/symptom in line with any overt connective tissue disease. In detail, she presented with no further clinical or instrumental sign or symptom of systemic lupus erythematosus (SLE) or Sjögren’s syndrome.

**Fig. 2:**
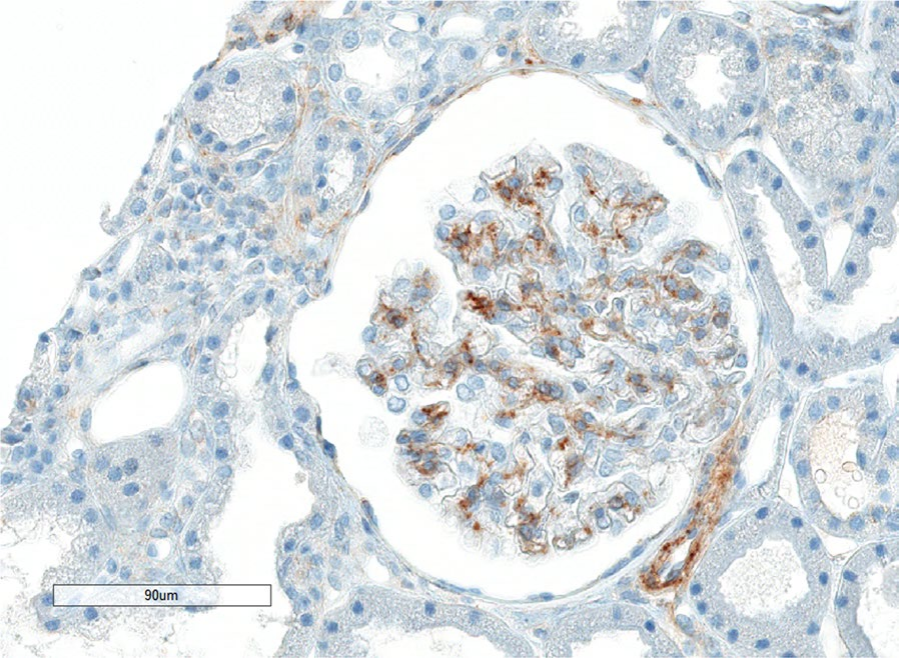
Ro52 immunohistochemistry. Granular positivity in mesangium and along rare glomerular capillaries. Controls biopsy from patients with lupus nephritis and SSA serological positive antibodies resulted in negative at immunohistochemistry for Ro52

In order to achieve a better characterization of the masked deposit, a immunochemistry investigation for SSA/52 and SSA/60 was performed [recombinant Human 60 kDa SS-A/Ro ribonucleoprotein protein (3-535AA) and Anti-Ro/SSA-Anti-Ro52 Sigma Sigma-Aldrich Co. LLC] (shown in Fig. 2). Kidney samples for patients with lupus nephritis and serological persistent antibodies against SSA/52 and SSA/60 were used as controls.

## Discussion and conclusions

*"When you hear hoofbeats behind you, don't expect to see a zebra" (Prof. Theodore Woodward)*

The MGMID is a recently discovered histopathologic pattern of glomerulonephritis characterized by subepithelial and mesangial deposits of IgG kappa detectable in paraffin immunofluorescence.

Some considerations are worth noting from our experience with this patient.

Firstly, as initially speculated, MGMID seems to be generally present in young females with autoimmune positive tests. Since their first description [1], it seemed evident that a good percentage of these patients present with some clinical signs and symptoms in line with a connective tissue disease, yet not sufficient for a definite diagnosis of conditions such as SLE or Sjögren’s syndrome. Patients with these profiles are commonly seen in the rheumatologic outpatients and are usually characterized by a benign prognosis [5]. However, in this setting, the new identification of urinary abnormalities may pose some diagnostic challenges. Our case confirmed the concept that the identification of persistent urinary abnormalities in patients with a suspected connective tissue disease should always support a prompt diagnostic workout and kidney biopsy should always be performed if not contraindicated [6].

Secondly, our patient presented with high titers of SSA, as often seen in Sjögren’s syndrome and SLE. To date, only one case of a patient with overt diagnosis of Sjögren’s syndrome with MGMID has been described. However, a pathogenic role for SSA in inducing renal manifestation has never been speculated [7]. Interestingly, glomerular involvement is less frequently observed than tubular disease in Sjögren’s syndrome [8]. When referring to glomerular lesions, membranoproliferative glomerulonephritis is the most frequently reported. Other glomerular diseases have also been described, to include minimal change disease, IgA nephropathy, focal segmental glomerulosclerosis, membranous nephropathy, fibrillary GN and vasculitis. In MGMID, unlike PLA2R-associated membranous glomerulopathy, deposits are subepithelial and mesangial in almost the totality of the reported cases. Importantly, incomplete involvement of the glomerular capillary walls is usually observed, suggesting that the Igs depositing in the glomeruli are more likely to be circulating immune complexes trapped in the subepithelial space rather than be related to in situ immune complex binding to podocyte antigens. This is also supported by the frequent finding of large hump-like deposits, which are known to be the consequence of trapping immune complexes from circulation. While only future prospective studies will clarify if SSA may have a pathogenic role in MGMID or if the presence of anti-RO antibodies represent only an ephimenomen, in this case we described for the first time by Ro52 immunohistochemistry a granular positivity in mesangium and along rare glomerular capillaries, potentially paving the way for a better understanding of this condition.

Finally, our patient did not experience a worsening of the clinical setting after more than 24 months of follow-up. Outcomes of MGMID are heterogeneous ranging from spontaneous remission to progression to end stage renal disease, making a better characterization of the patients’ profile highly needed to guide therapeutic choices.

With MGMID now increasingly identified as a novel clinical entity, international efforts are needed to better understand prognostic factors associated with different renal outcomes.

## Data Availability

All data generated or analyzed during this study are included in this article. Further enquiries can be directed to the corresponding author.

## Abbreviations

MN: Membranous nephropathy
MGMID: The membranous-like glomerulopathy with masked IgG kappa deposits pattern
ANA: Antinuclear antibodies
SAP: Serum amiloid P
PTAH: Phosphotungstic acid haematoxylin
APG: Apigenin
PLA2R: Antibodies to phospholipase A2 receptor
